# Antimalarial and antimicrobial activities of 8-Aminoquinoline-Uracils metal complexes

**DOI:** 10.17179/excli2016-101

**Published:** 2016-02-18

**Authors:** Kamonrat Phopin, Nujarin Sinthupoom, Lertyot Treeratanapiboon, Sarun Kunwittaya, Supaluk Prachayasittikul, Somsak Ruchirawat, Virapong Prachayasittikul

**Affiliations:** 1 Center for Research and Innovation, Faculty of Medical Technology, Mahidol University, Bangkok 10700, Thailand; 2 Department of Clinical Microbiology and Applied Technology, Faculty of Medical Technology, Mahidol University, Bangkok 10700, Thailand; 3 Center of Data Mining and Biomedical Informatics, Faculty of Medical Technology, Mahidol University, Bangkok 10700, Thailand; 4 Laboratory of Medicinal Chemistry, Chulabhorn Research Institute, and Program in Chemical Biology, Chulabhorn Graduate Institute, Bangkok 10210, Thailand; 5 Center of Excellence on Environmental Health and Toxicology, Commission on Higher Education (CHE), Ministry of Education, Thailand

**Keywords:** aminoquinoline, metal complexes, uracils, antimicrobial, antimalarial

## Abstract

8-Aminoquinoline (8AQ) derivatives have been reported to have antimalarial, anticancer, and antioxidant activities. This study investigated the potency of 8AQ-5-substituted (iodo and nitro) uracils metal (Mn, Cu, Ni) complexes (**1-6**) as antimalarial and antimicrobial agents. Interestingly, all of these metal complexes (**1**-**6**) showed fair antimalarial activities. Moreover, Cu complexes **2** (8AQ-Cu-5Iu) and **5** (8AQ-Cu-5Nu) exerted antimicrobial activities against Gram-negative bacteria including *P. shigelloides* and *S. dysenteriae*. The results reveal application of 8AQ and its metal complexes as potential compounds to be further developed as novel antimalarial and antibacterial agents.

## Introduction

Aminoquinoline, a class of heterocyclic scaffold with an amino group, is frequently found in diverse bioactive compounds. Aminoquinoline derivatives have a variety of pharmacological properties including antimalarial, antileishmanial (Kulshrestha et al., 2011[17]), antitrypanosomal (Yardley et al., 2010[42]), anticancer, antibacterial (Jain et al., 2005[11]; Kaur et al., 2007[15]), and antifungal (Kaur et al., 2011[14]) activities, as well as metal chelator (Nguyen et al., 2015[24]). 8-Aminoquinolines, the first group of synthetic compounds, have been synthesized for antimalarial activity. 

**Figure 1:**
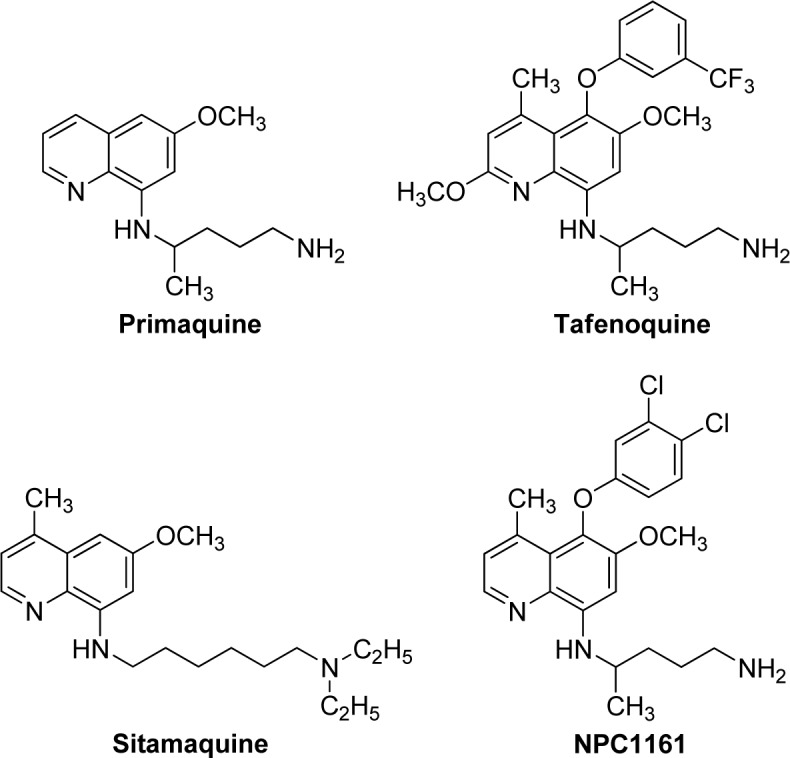
Chemical structures of primaquine, tafenoquine, sitamaquine and NPC1161

Primaquine, 8-aminoquinoline (8AQ) analog, is approved by the Food and Drug Administration (FDA) for the treatment of relapses in *Plasmodium* infections (Hill et al., 2006[10]). It exhibits antimalarial activity against *P. vivax*, *P. ovale* and *P. falciparum* (White et al., 2014[41]). In addition, tafenoquine is the 8AQ drug being developed for *P. vivax* that is currently in clinical trials (Nasveld et al., 2010[23]). However, the usage of these 8-aminoquinolines is limited because they are likely to cause red blood cell hemolysis in patients with glucose-6-phosphate dehydrogenase (G6PD) deficiency (Bolchoz et al., 2001[3]). Regarding safety treatment and prevention of hemolytic anemia, patients with malarial infection should be screened for G6PD deficiency before treating with these medicines. Moreover, 8-aminoquinolines (sitamaquine and NPC1161) have been shown to be active against *Leishmania* and *Trypanosoma* parasites (Kulshrestha et al., 2011[17]; Yardley et al., 2010[42]). Thus, searching for novel potential bioactive compounds is required for the treatment of malaria-infected patients. These drugs/compounds (Figure 1(Fig. 1)) are 8-AQ derivatives in which their 8-NH_2_ groups are substituted with various alkylamines side chain (Yardley et al., 2010[42]; Jain et al., 2005[11]; Kaur et al., 2007[15]) including bis (8-AQs) (Kaur et al., 2011[14]).

Metal ions play important roles in many biological processes in living organisms. Metal complexes constitute a central metal atom surrounded by ligands (Cox, 2005[7]), showing significant progress in treatment of human diseases including cancer, leukemia, infection and inflammation (Rafique et al., 2010[30]). Metal complexes have been reported to deliver ligands as drugs to target sites, leading to reduce side effects and improve pharmacokinetics. Recently, metal complexes of 8-hydroxyquinoline (8HQ) have been shown to be antiviral, antiparasitic, antioxidant, antiinflammatory, and antidiabetic agents (Prachayasittikul et al., 2013[29]). Quinoline-based compounds have high selectivity of human malaria due to their metal binding/chelating abilities and lipophilicities to penetrate cell membranes to reach the target sites of action (Scheibel and Adler, 1982[34], 1980[35], 1981[33]). 

The activation by redox active metal ions, Cu(II), Fe(III), and Zn(II), was observed in patient’s brains with Alzheimer’s disease (AD) (Nguyen et al., 2014[25]). These metal ions can bind strongly with beta amyloid in amyloid plaques which cause oxidative stress, reactive oxygen species (ROS) and beta-amyloid toxicity (Nguyen et al., 2015[24]). Additionally, the excess of copper associated with abnormal tau phosphorylation in the brain can induce neuronal inflammation and death. Tetradentate ligands, clioquinol and 8AQ are known as metal ion chelators, which can transport copper within metal deficient neurons for the treatment of AD (Ceccom et al., 2012[6]). One of bis (8-aminoquinoline) ligands, PA1637, has high selectivity for copper (II) chelation used in a model of non-transgenic mice injected with Ab1-42 oligomer via an intracerebroventricular area. It can prevent the memory loss in the mouse model (Nguyen et al., 2014[25]).

Recent reports have demonstrated that chemical structure modifications of 8AQ mostly improve antimalarial activity (Kannan et al., 2015[12]; Miranda et al., 2014[22]) and decrease toxicity (Kaur et al., 2012[13]). Additionally, transition metal complexes of mixed ligands 8AQ-uracil derivatives (5-iodo and 5-nitro) have been reported to display their antioxidative and cytotoxic activities by our group (Pingaew et al., 2013[27]). Various drugs and bioactive compounds such as 8HQ have been found to exert anticancer and antimalarial activities (Prachayasittikul et al., 2013[29]). Malaria parasites, *P. falciparum*, exhibit rapid nucleic acid synthesis during their intraerythrocytic growth phase which requires robust supplies of purine and pyrimidine in the metabolic pathways (Cassera et al., 2011[5]). Orotic acid is the only preformed pyrimidine utilized by malarial parasite (Rathod et al., 1992[32]). 5-Fluoroorotate, pyrimidine derivative, is recognized to have antimalarial activity against *P. falciparum* with IC_50_ < 10 nM (Cassera et al., 2011[5]). Other pyrimidine derivatives such as uracil and 5-fluorouracil have been reported to show weak inhibition of *P. falciparum *with IC_50 _range of 5-10 µM (Cassera et al., 2011[5]). Thus, a combination of 8AQ and uracils (5-iodouracil and 5-nitrouracil) in the one molecule as metal-based compounds may provide the compounds with enhanced antimalarial activity. Here, the potential of metal complexes of 8AQ-5-substituted uracils as antimalarial and antimicrobial agents has been reported.

## Materials and Methods

### Tested compounds

**Figure 2:**
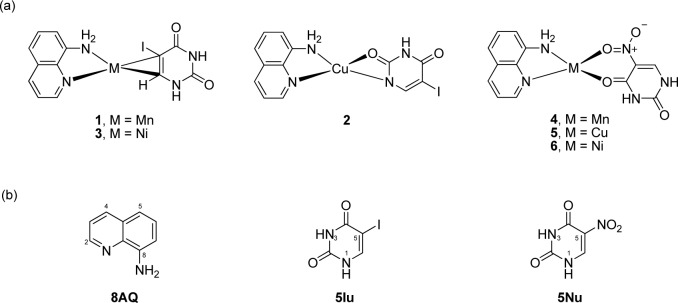
Chemical structures of (a) 8AQ-5Iu (or 5Nu) metal complexes and (b) ligands

8AQ metal (Mn, Cu, Ni) complexes (**1-6**, Figure 2a(Fig. 2)) were synthesized and confirmed by spectral data by our group (Pingaew et al., 2013[27]). Chemical structures of free ligands 8AQ, 5-iodouracil (5Iu), and 5-nitrouracil (5Nu) are shown in Figure 2b(Fig. 2).

### Parasite strain and in vitro culture

*P. falciparum* (K1 strain), chloroquine resistance, was maintained following the previous method (Trager and Jensen, 1976[40]). *P. falciparum* was cultured in RPMI-1640 medium containing 25 mM of HEPES, 10 % of human serum, and 40 mg/L of gentamicin. Sorbitol was used to synchronize *P. falciparum* before starting the assay following the previous method to gain ring stage of malaria (Lambros and Vanderberg, 1979[18]). To avoid sorbitol effect, the culture was incubated for 48 h before treatment with the compounds.

### Antimalarial assay 

Antimalarial activities of the tested compounds (**1-6** and ligands) were investigated based on the rate of *P. falciparum* growth via microscopic technique using methanol-fixed Giemsa stain. The ring stage-infected RBCs were diluted with fresh uninfected RBCs and complete medium to obtain a final concentration at 2 % parasitemia. The parasite suspension was put onto a 96-well microtiter plate, then the microtiter plate was added with different final concentrations of each compound as follows: 0.01, 0.1, 1, 10, 100 and 1000 µg/mL compared to a reference drug; artesunate 0.2 ng/mL (IC_50_). The rate of *P. falciparum* growth was observed for 4 days, and each concentration was performed in triplicate. The efficacies of the compounds were evaluated by determining the concentration that reduced the growth of parasite by 50 % (IC_50_).

### Antimicrobial testing 

Method of agar dilution was performed to study the antimicrobial activity as formerly described (Prachayasittikul et al., 2011[28]). All compounds were dissolved in dimethyl sulfoxide (DMSO), and were separately mixed with 1 mL of Mueller Hinton (MH) broth. Then, the final concentrations of 32-256 µg/mL were carried out by transferring the mixture to the MH agar, and the negative control was MH broth. The cell concentration of microbes used in this study was adjusted to 10^8^ cells/mL in 0.9 % normal saline after the microbes were cultured at 37 °C for 24 h in the MH broth. The inhibitions of microbial growth were detected in each compound following the inoculation onto the MH agar, and incubation at 37 °C for 24 h. Twenty-four bacteria and two yeasts were tested as listed in Table 1(Tab. 1). 

## Results and Discussion

### Antimalarial activity 

The synthesized 8AQ metal complexes (**1-6**) and free ligands were tested against chloroquine-resistant *P. falciparum *(K1) using artesunate as a reference drug. It was found that all of the metal complexes showed fair antimalarial activity with IC_50_ 100-1000 µg/mL (Table 2(Tab. 2)). Similarly, the free ligands (8AQ, 5Iu and 5Nu) of such metal complexes exhibited antimalarial activity with the same IC_50_ values. To date, the antimalarial activity of these metal complexes (**1-6**) as well as 8AQ, 5Iu and 5Nu has not been reported in the literature. Quinoline-based compounds are potent chelators having high lipophilic property. The antimalarial activity of these compounds was exhibited by inhibiting the growth of parasites and glycolysis process due to the inactivation of various enzymes including metalloprotein oxidase (Scheibel and Adler, 1980[35]). The antimalarial activity of 8AQ may be derived from its formation of quinoneimine metabolite which ultimately generates hydrogen peroxides and oxidative stress in erythrocytes (Shiraki et al., 2011[37]). It is reasonable to suggest that the dissociation of lipophilic 8AQ-metal complexes (**1-6**) could give rise to charged complexes (8AQ-M)^+^/ or (5Iu/5Nu-M)^+^, and free ligands (8AQ, 5Iu and 5Nu) (Pingaew et al., 2013[27]) in which the 8AQ chelator can interact with parasitic enzyme in different ways such as interacting with SH or NH_2_ group, and with certain metal ion of the enzyme leading to the growth inhibition of *P. falciparum* (Owens, 1953[26]).

As the aforementioned that orotic acid (uracil-6-carboxylic acid) is required for intraerythrocytic growth of *P. falciparum* (Rathod et al., 1992[32]). Orotic acid derivative, 5-fluoroorotate, has been reported to show antimalarial activity against the *P. falciparum* (Cassera et al., 2011[5]). In addition, uracil and 5-fluorouracil displayed weak antimalarial activity (Cassera et al., 2011[5]). In this regard, antimalarial activity of 5Iu and 5Nu may be possibly involved in the pyrimidine metabolic pathway of malaria parasite (Rathod et al., 1992[32]). Another plausible explanation may be due to the electron donor property of 5Iu and 5Nu (Pingaew et al., 2013[27]) that could interact with certain metal ions of the parasitic enzyme (Owens, 1953[26]). 

### Antimicrobial activity 

The agar dilution method (Prachayasittikul et al., 2011[28]) was performed to determine the activity of 8AQ metal complexes (**1**-**6**), 5Iu, 5Nu, and 8AQ, as antimicrobial agents against twenty-six microbes. DMSO was used as a control. The results revealed that only Cu-complexes **2 **and **5** inhibited the growth of Gram-negative bacteria (Table 3(Tab. 3)), and the DMSO showed no effect toward the tested microorganisms. The Cu-complex** 5 **exhibited antigrowth activity against *P. Shigelloides *with MIC value of 256 µg/mL. In addition, the *P. shigelloides* was partially inhibited by compound **5** at 128 µg/mL and 64 µg/mL showing 75 % and 25 % inhibition, respectively. The Cu-complex** 2** at 256 µg/mL exerted 25 % inhibition against *P. shigelloides* and *S. dysenteriae*. Other metal complexes (**1**,** 3**,** 4** and **6**) were found to be inactive antimicrobials. It should be noted that the free ligands (8AQ, 5Iu and 5Nu) displayed no antimicrobial activity. This might be suggested that the lipophilic property of Cu-complexes **2** and **5** deriving from complexation of mixed ligands, 8AQ-5Iu and 8AQ-5Nu, could enhance absorption of compounds to their target sites of action. It was observed that the metal complex **5** showed better antimicrobial activity as compared to the complex **2**. It could be reasonably explained that the higher electron withdrawing group (5-nitro) of 5Nu ligand facilitated the better dissociation of the complex **5** to give charged complex (8AQ-Cu)^+^ and free ligand (5Nu) comparing to the complex** 2** with lower electron withdrawing group (5-iodo). The charged complex (8AQ-Cu)^+^ will interact and block the metal binding on the enzyme accounting for antimicrobial activity of the metal complexes (Anjaneyulu et al., 1982[1]; Srisung et al., 2013[38]). 

*P. shigelloides* is a Gram-negative bacterium commonly found in fresh and brackish water (Farmer et al., 2006[8]), soil and animals such as fish, oyster, mussel, prawn and crab as well as in human (Khardori and Fainstein, 1988[16]). *P. shigelloides* is an opportunistic pathogen which is associated with gastroenteritis (Mandal et al., 1982[20]; McNeeley et al., 1984[21]; Schubert and Holz-Bremer, 1999[36]; Stock, 2004[39]). This bacterium is considered as one of the important causes of traveler's diarrhea (Schubert and Holz-Bremer, 1999[36]). Moreover, it was reported to cause extraintestinal infection with high fatality rate such as bacteremia, meningitis, and pseudoappendicitis (Billiet et al., 1989[2]; Brenden et al., 1988[4]; Fischer et al., 1988[9]; Lee et al., 1996[19]). *P. shigelloides* has been found to resist to several antimicrobial drugs including penicillin, vancomycin, tetracycline and erythromycin, especially cabapenems, fluoroquinolones, trimethoprim and cotrimoxazole (trimethoprim-sulpha-methoxazole) (Ramalivhana and Obi, 2009[31]). 

## Conclusion

Oxidative stress is involved in the pathophysiology of malarial infection. It is well recognized that *Plasmodium* parasites digest hemoglobin resulting in the production of heme which triggers the generation of reactive oxygen species, and can lead to anemia and death. Thus, compounds with antioxidant activity may alleviate the progression of malarial infection, and possibly prevent the sequelae. In this study, 8-aminoquinoline metal complexes become an interesting class of bioactive compounds as antimalarial against *P. falciparum*, and antimicrobial against Gram-negative bacteria. Moreover, these metal complexes (8AQ-5-substituted uracils) are more effective antimicrobials than 8AQ. 

It can be concluded that 8- aminoquinoline and its metal complexes show a wide range of antimalarial and antimicrobial activities. Therefore, further investigation will be explored to understand and to achieve the compounds with improved activities. 

## Notes

Supaluk Prachayasittikul and Virapong Prachayasittikul (Department of Clinical Microbiology and Applied Technology, Faculty of Medical Technology, Mahidol University, Bangkok 10700, Thailand; email: virapong.pra@mahidol.ac.th) contributed equally as corresponding authors.

## Conflict of interests

The authors have declared that no competing interests exist.

## Acknowledgements

This project is supported by the Office of the Higher Education Commission, Mahidol University under the National Research Universities Initiative and Annual Government Grant of Mahidol University (2556-2558 B.E.).

## Figures and Tables

**Table 1:**
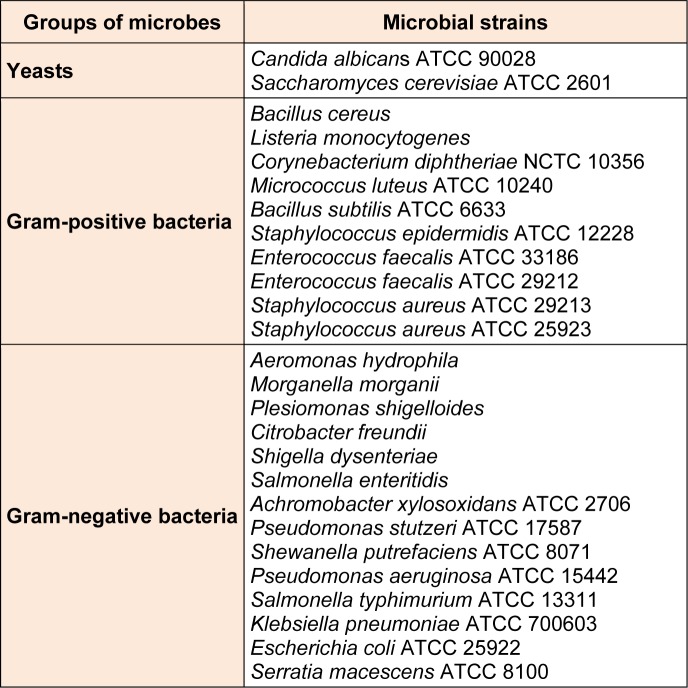
Twenty-six microorganisms used in antimicrobial testing

**Table 2:**
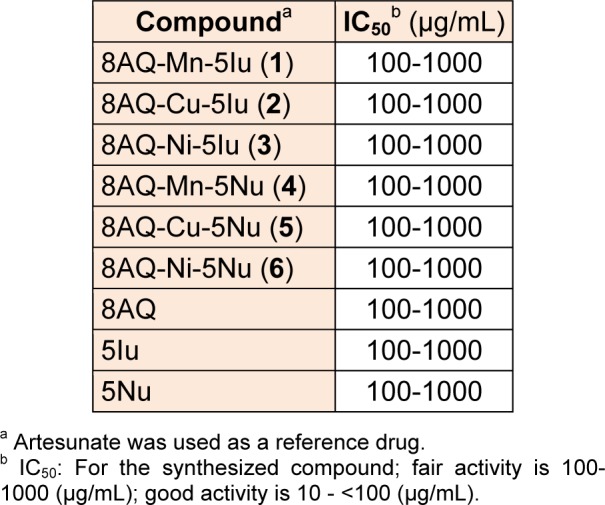
Antimalarial activity of metal complexes (1-6) and ligands

**Table 3:**
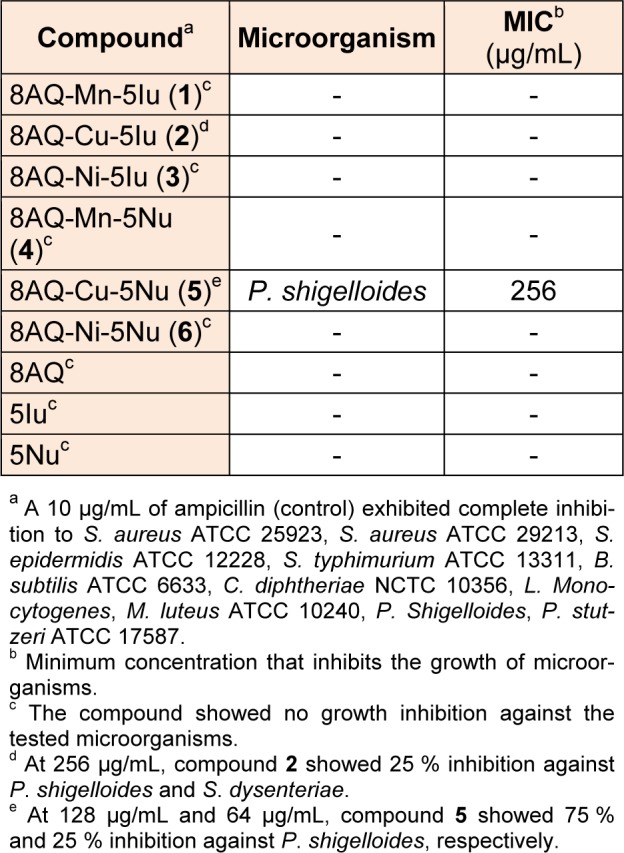
Antimicrobial activity of metal complexes (1-6) and ligands
